# Supplementary material for: The Potential of a New Natural Vessel Source: Decellularized Intercostal Arteries as Sufficiently Long Small-Diameter Vascular Grafts
Source: Bioengineering (Basel). 2024 Jul 10;11(7):700. doi: 10.3390/bioengineering11070700 (PMC11273892; doi:10.3390/bioengineering11070700)
Supplement: Supplementary file 1 [file bioengineering-11-00700-s001.zip › bioengineering-3010824-supplementary.pdf]

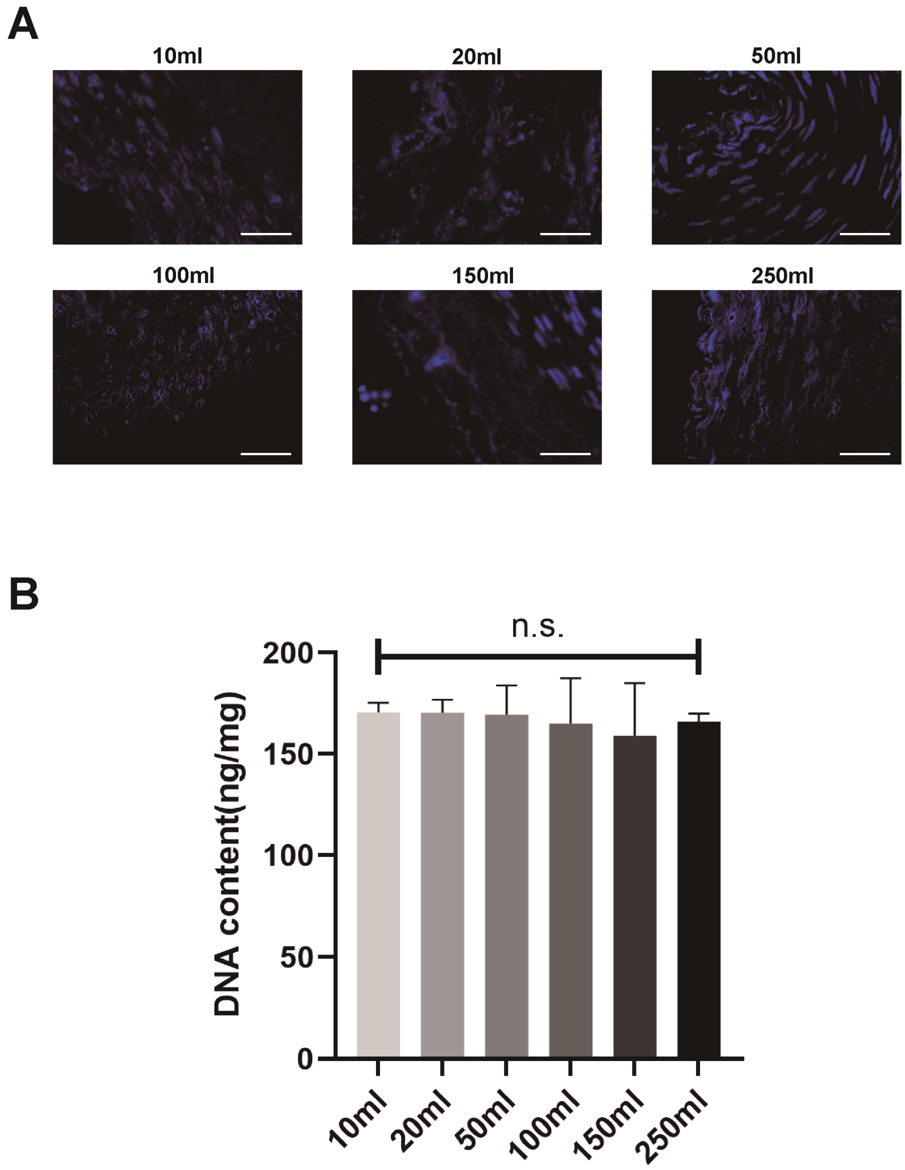

Figure S1. Evaluation of Decellularization Effectiveness (**A**) DAPI staining of bovine intercostal arteries decellularized by different flow rate:10ml, 20ml, 50ml, 100ml, 150ml, 250ml, processing times are 24 hours. scale bar is 50μm (**B**) DNA content for each group. The test results for each animal are presented as 10ml, 20ml, 50ml, 100ml, 150ml, 250ml group. All processing times are 24 hours. "n.s." indicates no significant differences between comparison groups.

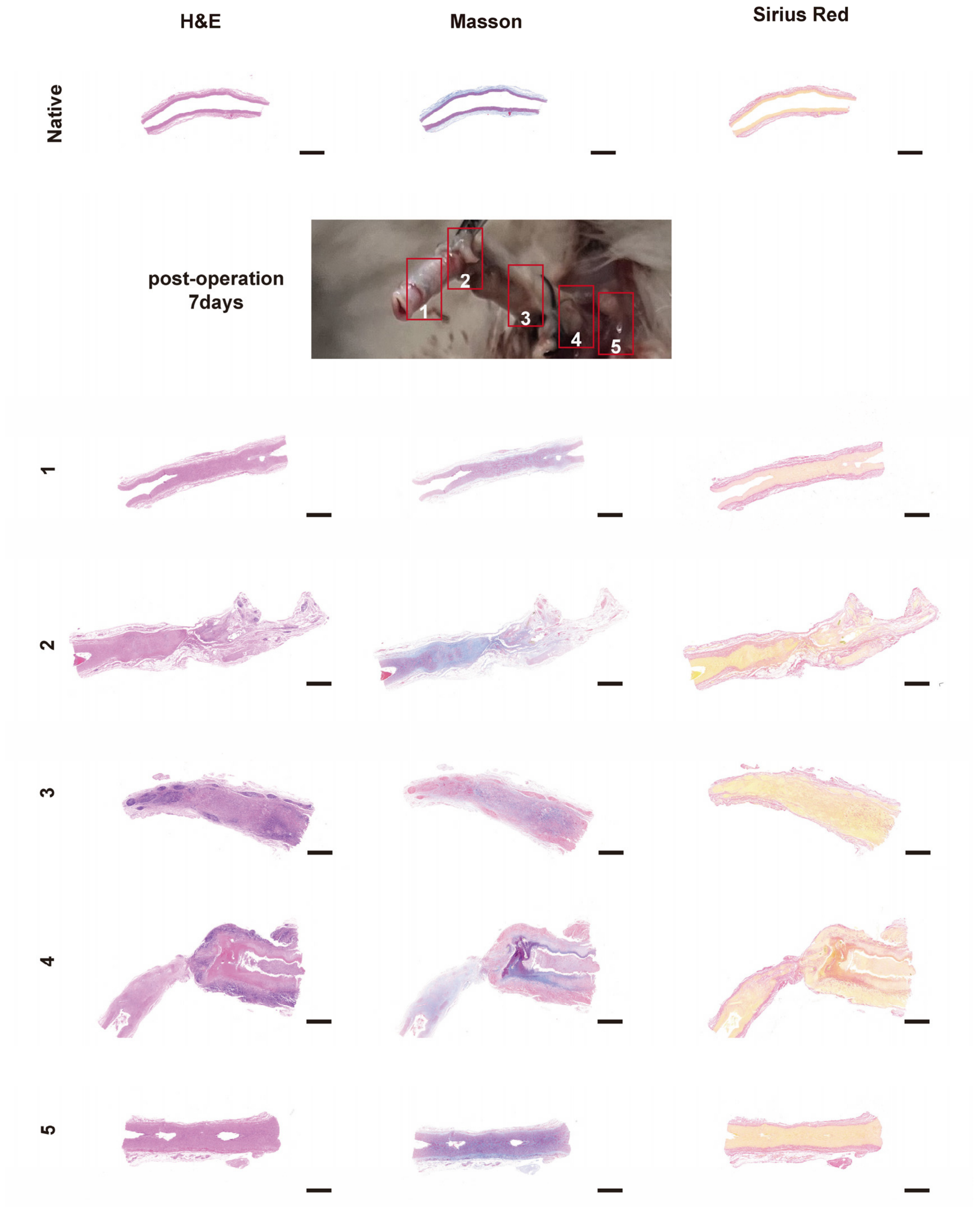

Figure S2. Histological sections of BDIA-transplanted rabbit carotid artery, seven days post-transplantation. Native: Contralateral carotid artery of the rabbit. 1. Distal native artery of the operated side. 2. Distal anastomosis site. 3. BDIAs. 4. Proximal anastomosis site. 5. Proximal native artery. Scale bar: 1000μm.Taking into account the characteristics of the bovine intercostal artery (BDIA) that are more suitable for clinical coronary artery bypass grafting (CABG), we conducted in vivo experiments using the rabbit carotid artery transplantation model to evaluate its biocompatibility. However, the experimental results indicated that BDIAs failed to meet the requirements. Ultrasound monitoring of blood flow post-transplantation revealed no blood flow in the operated carotid artery on day 7. Subsequently, the experimental animals were euthanized for dissection, where macroscopic examination showed stenosis, and pathological sections revealed thrombosis formation .
